# MOF-derived multifractal porous carbon with ultrahigh lithium-ion storage performance

**DOI:** 10.1038/srep40574

**Published:** 2017-01-11

**Authors:** Ang Li, Yan Tong, Bin Cao, Huaihe Song, Zhihong Li, Xiaohong Chen, Jisheng Zhou, Gen Chen, Hongmei Luo

**Affiliations:** 1 State Key Laboratory of Chemical Resource Engineering, Beijing Key Laboratory of Electrochemical Process and Technology for Materials, Beijing University of Chemical Technology, Beijing, 100029, P. R. China; 2 Beijing Synchrotron Radiation Facility, Institute of High Energy Physics, Chinese Academy of Sciences, 19B Yuquan Road, Beijing 100049, P. R. China; 3 Department of Chemical and Materials Engineering, New Mexico State University, Las Cruces, New Mexico 88003, United States

## Abstract

Porous carbon is one of the most promising alternatives to traditional graphite materials in lithium-ion batteries. This is not only attributed to its advantages of good safety, stability and electrical conductivity, which are held by all the carbon-based electrodes, but also especially ascribed to its relatively high capacity and excellent cycle stability. Here we report the design and synthesis of a highly porous pure carbon material with multifractal structures. This material is prepared by the vacuum carbonization of a zinc-based metal-organic framework, which demonstrates an ultrahigh lithium storage capacity of 2458 mAh g^−1^ and a favorable high-rate performance. The associations between the structural features and the lithium storage mechanism are also revealed by small-angle X-ray scattering (SAXS), especially the closed pore effects on lithium-ion storage.

The increasing demands for high-performance power batteries have gained a sustained research and development in lithium-ion battery electrodes^1^,^2^. Up to date, several mechanisms of lithium storage in pure carbon materials have been proposed, which can be roughly classified into three types according to the lithium embedded position: the intercalation into graphite layer structures, the storage at interfaces and surfaces and the storage in defects with different dimensionality^3^. The disordered carbons derived from certain polymers can provide an excess storage capacity by the additional bulk intercalation of lithium atoms, which corresponds to the formation of Li_2_C_6_ and Li_3_C_6_. The presence of covalent Li_2_ molecules^3^,^4^ is usually used to explain the unexpected high capacity for bulk storage (even up to 1116 mAh g^−1^). In some reports, the models of “house of cards” and “falling cards” were proposed to explain the extra lithium ion storage at the external and internal interfaces of the disordered carbons^5^,^6^. Surface stored lithium can be treated as an intermediate state between intercalated lithium and metallic lithium, which achieves by the occupancy of various lithium sites to form lithium multilayers on the surface of carbon^7^. Another important mechanism considering the constructive role of defects^3^ is of the view that the microstructures, like cavities, nanopores and vacancies, can also bring an extra capacity^8^,^9^. The pore-filling model presumes that the pores in the carbon materials, if small enough, will be filled with adsorbed lithium atoms^8^. These nanopores will be accommodated by clusters of lithium atoms in which the bond is weaker than that in metallic lithium, while solvent molecules cannot enter the pores. An interesting phenomenon in this model is that the closed pores are accessible for lithium with fast permeability but not for N_2_ molecules, which makes lithium storage sites of this type cannot be characterized by N_2_ sorption method^3^; therefore, there are still no experimental evidence to support such an instructive viewpoint. For all the aforementioned mechanisms, the structures of carbon materials, which depend mainly on the types of precursors and conditions of pyrolysis, have a great effect on the lithium storage modes^10^.

In order to break through the limitation of the intercalation capacity of the commercial graphite materials (372 mAh g^−1^) and the organic precursor-derived carbon, significant efforts have been made to design novel materials to enhance the reversible lithium storage capacity by increasing the degree of lattice disorder, enlarging surface area and increasing defect density^3^,^11^,^12^. Porous carbon can satisfy these structural requirements almost simultaneously^11^. In recent years, a kind of porous carbon materials which have hierarchical pore structures with a broad pore size distribution have been designed to optimize the conditions for electrolyte permeation and lithium accommodation, which are aroused tremendous interest^13^. These hierarchical porous carbon materials exhibit an enhanced electrochemical performance than the traditional porous carbon materials^14^. Inspired by the concepts of fractal structure and hierarchical pore size distribution, the structure design of porous carbon, by organizing the hierarchical pores into porous and surface fractal structures, can be introduced to take full advantage of all the lithium storage modes^15^.

Metal-organic frameworks (MOFs) are a kind of promising precursors for porous carbon preparation, due to their tunable ordered porous structures and compositions^2^,^16^,^17^,^18^. Many works prepared porous carbon from MOFs for the applications in supercapacitors^19^,^20^,^21^, lithium-sulfur batteries^22^,^23^, oxygen reduction reactions^19^,^24^,^25^,^26^, as well as lithium-ion batteries (LIBs)^2^,^27^,^28^,^29^,^30^. Their highly porous structures and heteroatom effects have led to unexpected performances in these fields. However, as electrodes for LIBs, most reports concentrated on introducing heteroatoms to enhance the lithium storage performance, rather than the structure design and architecture assembly. This study aims to design and prepare hierarchical porous carbon materials with multifractal structures for high-performance LIBs by varying the conditions of the precursor pyrolysis process. The relationships between the structural features and the lithium storage mechanisms are also investigated. In addition, the important role of closed pores in lithium storage will be revealed by a comprehensive analysis of porous properties of products using synchrotron radiation small-angle X-ray scattering (SAXS).

## Results and Discussion

**Figure 1:**
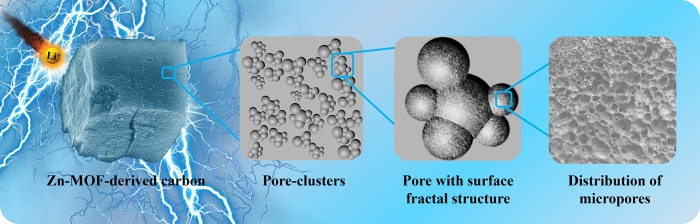
Schematic illustration of the multifractal structure of MOF-derived porous carbon (FPC and VFPC).

**Figure 2:**
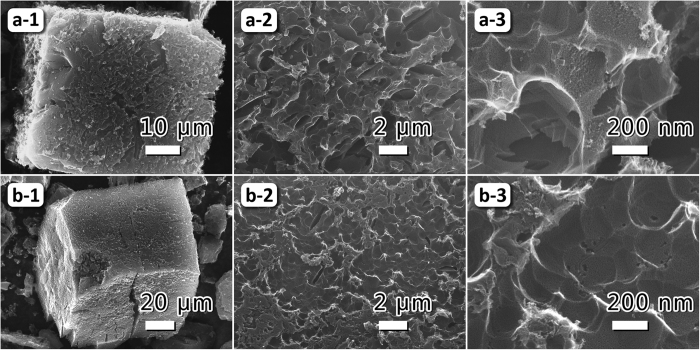
Morphology characterizations of FPC and VFPC by SEM. (**a**) and (**b**) are images of FPC and VFPC, respectively.

**Figure 3:**
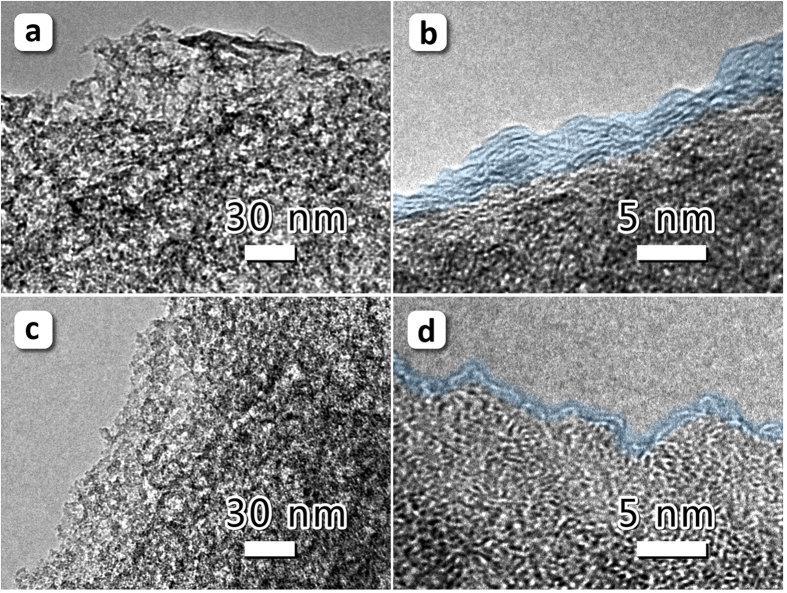
Microstructural characterization of FPC and VFPC. (**a,b**) and (**c,d**) are HRTEM images of FPC and VFPC, respectively. No obvious ordered structure can be found, indicating the formation of amorphous carbon. As highlighted in blue in (**b**) and (**d**), VFPC shows a more irregular surface structure than that of FPC.

A Zn-based MOF (Zn-MOF) with terephthalic acid as ligand was used as precursor, which exhibits a high Brunauer-Emmet-Teller (BET) specific surface area and a relatively wide pore size distribution (see Supplementary SI.1). A simply thermal treatment was used to design and prepare porous carbon with multifractal structures, as illustrated in Fig. 1, by varying the pyrolytic pressure. After pyrolysis, both of the products have maintained the original morphology of the shape of Zn-MOF grains (Fig. 2 for products; Fig. S1c & d for precursor); however, the surfaces of the products are cavernous. As can be seen in Fig. 2, the pores and cavities in the sample pyrolyzed in vacuum (VFPC) are smaller and denser than those of the sample pyrolyzed under normal pressure (FPC), and all the pores and cavities are globulous and interconnected. The results of XRD and EDS show that both FPC and VFPC are amorphous carbon (Fig. S2 for EDS; Fig. S3a for XRD). XRD patterns of FPC and VFPC both display two weak diffraction peaks at 2θ = 23.7° and 43.9°, corresponding to (002) and (100) reflections of carbon, respectively. None of the original ordered porous structures in Zn-MOF are maintained after the pyrolysis process, implying the collapse of carbon skeletons. VFPC shows a higher purity of carbon but a lower carbon yield based on MOF precursor (VFPC, 2.19%; FPC, 2.26%), indicating that the vacuum atmosphere can improve the gasification reactions and purify the carbon materials. In Raman spectroscopy (Fig. S3b), the characteristic peaks are typically indicated to elemental carbon. The band intensity ratio of I_D_/I_G_ for the D band and the G band is a significant and widely used factor to quantify the disorder degree of carbon materials^2^. Based on the data in Fig. S3b, we can calculate that the I_D_/I_G_ for FPC and VFPC are 1.14 and 1.11, respectively. It can be concluded that the disordered structure in FPC is higher than that in VFPC. The microstructures of samples were investigated by HRTEM in detail, as illustrated in Fig. 3a–d. Both FPC and VFPC show a porous structure, and their local magnification pictures displayed in Fig. 3c,d show a typical characteristic of amorphous carbon with random and disordered lattice fringes. The surface of VFPC is more irregular than that of FPC, which might be ascribed to the strong etching effect and pyrolysis process in vacuum.

**Figure 4:**
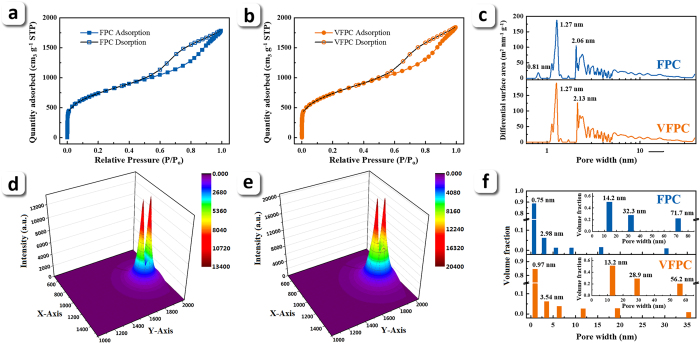
Porous property characterizations of FPC and VFPC. (**a**) and (**b**) are N_2_ adsorption isotherms of FPC and VFPC, respectively; (**c**) is the pore size distribution of the samples. (**d**) and (**e**) are 2D SAXS pattern with transmitted intensity as a Z-axis for FPC and VFPC, respectively. The color bars are the scaling of intensity. (**f**) Is the pore size distribution of the samples, which was determined by a cascade tangent rule of Jellinek method (Fig. S3). The insets in (**f**) are the pore size distribution of the fully lithiated samples, respectively.

The pore properties of the two samples were investigated by two techniques independently: N_2_ sorption and SAXS. The N_2_ adsorption isotherms of the two samples both illustrate characteristics of type IV (Fig. 4a,b), indicating the existence of mesopores^31^. The rapid uptake of adsorption curves at low relative pressure range (P/P_0_ < 0.1) is ascribed to the adsorption of N_2_ in micropores^31^. As illustrated in Table 1, the BET specific surface area (S_BET_) and pore volume (V) of VFPC show slight bigger values than those of FPC, while the micropore volume (V_mic_) of VFPC is smaller. The pore size distributions of both samples, simulated by non-local density functional theory (NLDFT) model, show a wide distribution in the range of micropores and mesopores, indicating a hierarchical porous structure (Fig. 4c). The average pore size of VFPC and FPC simulated by BET method are 4.41 nm and 4.31 nm, respectively.

Further investigation was performed by SAXS to confirm the pore structure of the samples, including the properties of open pores and closed pores (Fig. 4d–f). The removal of the scattering background and the normalization of the scattering intensity have been made to correct the primary data. The specific surface area (S_SAXS_) from SAXS data can be obtained by the Porod’s law and the invariant (Fig. S4)^32^. The results show that the S_SAXS_ of FPC and VFPC are 2870 and 3125 m^2^ g^−1^, respectively. Significantly, the values of S_SAXS_ for both samples, especially for VFPC, are larger than those of S_BET_ obtained by N_2_ sorption method. Such phenomenon could be attributed to the constrictions that hinder the diffusion of N_2_ molecules, in some parts of closed pores and blind pores, or the open micropores that are too narrow to accommodate two layers of N_2_ molecules^33^. The average pore size of VFPC is 2.12 nm, which is still bigger than that of FPC (1.55 nm). The pore size distributions of the two samples from SAXS method also display hierarchical characteristics, as shown in the NLDFT results; however, the contribution of micropores to the total pore volume that calculated by the SAXS method is much higher than the results from N_2_ sorption method (Table 1, see Supplementary SI.3 & 4 for calculation details). As illustrated in Fig. 4f, the contributions of micropores to S_SAXS_ for FPC and VFPC are almost all the super-micropores (<1 nm). The most probable pore sizes of FPC and VFPC are 0.75 and 0.97 nm, respectively, which occupy the most of the scatterer volume, indicating the existence of a large amount of super-micropores that cannot be detected by N_2_ sorption. Across the two samples, considering the results of N_2_ sorption method and Raman spectra, we can conclude that the vacuum carbonization will expand the specific surface areas of the products, especially the contribution from micropores, and enlarge the average pore sizes but decrease the disordered degree of carbon.

Irregular geometric structures are also characteristics of the samples, which can be quantified by the fractal analysis with SAXS method^32^. The SAXS intensity from fractal samples obeys a power-law form, I = I_0_q^−α^, where α can be used to determine the fractal type and dimensions (Fig. S5)^32^. The analysis shows two types of fractal structures in the samples, porous fractal and surface fractal. The porous fractal behavior of both FPC and VFPC can be characterized by porous fractal dimension D_p_. D_p_ are 2.70 and 2.19 for FPC and VFPC, respectively, and the higher D_p_ means a larger degree of inhomogeneity in porous distribution of the two samples^32^. Moreover, the surface fractal behavior is observed and characterized by surface fractal dimension D_s_. D_s_ are 2.39 and 2.86 for FPC and VFPC, respectively. The surface irregularities of the two samples also can be confirmed in their HRTEM pictures in Fig. 3a–d. The bigger value of D_s_ of VFPC reveals a more irregular and rougher surface strucure^34^. In addition, the lnI-lnq curve of VFPC exhibits a surplus of surface fractal behavior, indicating a surface fractal geometric structure in a different scale. The VFPC shows a multifractal behavior in the organization of its hierarchical pores, suggesting that the vacuum pyrolysis can lead to more complex fractal structures.

Combining the results of SEM, SAXS and N_2_ sorption method, one can get a clear information on the porous structure of the samples. As illustrated in Fig. 1, the porous system is made up of “pore clusters” which are mainly comprised of mesopores. This is reflected as a porous fractal behavior in SAXS analysis. Most of the micropores are distributed on the surface of pores in “pore clusters” densely. The VFPC even shows a surface bi-fractal behavior in the distribution of its micropores.

**Figure 5:**
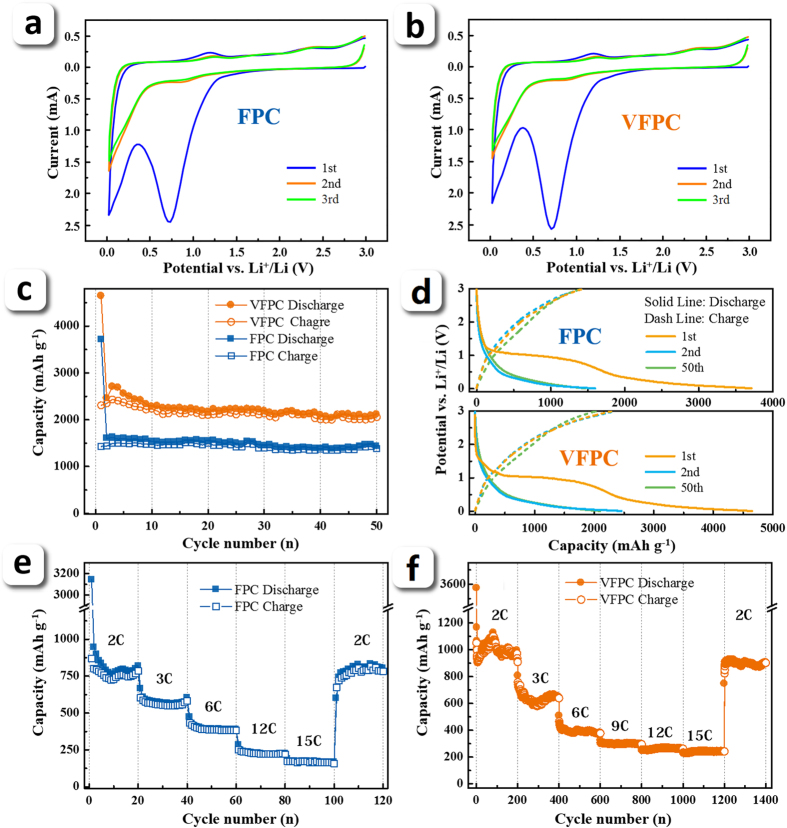
Electrochemical performance of FPC and VFPC. (**a**) Cycle performances of the samples at 0.2 C. (**b**) Is Galvanostatic charge-discharge profiles at 0.2C with a voltage range between 0.01 and 3.0 V for the 1st, the 2nd and the 50th cycle of FPC and VFPC, respectively; (**c**) and (**d**) are high-rate performances of FPC and VFPC at each rate, respectively. (**e**) and (**f**) are CV curves of FPC and VFPC, respectively.

Electrochemical performances of the as-products were measured with a two-electrode coin-type cell with lithium foil as counter electrode. The cyclic voltammetry (CV) results of the two samples show a typical porous carbon lithiation appearance (Fig. 5a,b). The first (0.01–0.3 V) and second (0.5–1.0 V) peaks can be attributed to the Li-insertion into carbon matrix, and the lithium ion storage on surface, respectively^2^,^3^. As can be seen in Fig. 5c, the first reversible capacity (Li-desertion) of FPC is 1600 mAh g^−1^, and that of VFPC even reaches as high as 2458 mAh g^−1^ at 0.2 C (1C = 370 mA g^−1^). After 50 cycles, the reversible capacity of VFPC is still at 2016 mAh g^−1^. The charge-discharge profiles in Fig. 5d show a plateau at approximately 1 V in 1st cycle which can be ascribed to the formation of SEI film on the electrode surfaces^2^, and the contribution of reversible capacity mainly occurs under 0.5 V. The cyclic voltammetry results of the two samples are consistent with charge-discharge profiles very well (Fig. 5a–d). Durable cycle life of FPC and VFPC at high rate is also obtained (Fig. 5e,f). The reversible capacity of VFPC at 2C is 1012 mAh g^−1^, which is ca. 50% of that at 0.2C. Even at a rate of 15C, a reversible capacity of 245 mAh g^−1^ is still maintained, and the tests show an excellent cyclic stability at each rate. For the first time, the fractal structure is used in porous carbon design for lithium storage, which can improve the lithium storage performance dramatically. The fractal porous structure can provide favorable high-rate performance and excellent cyclic stability by adjusting the pore spatial distribution in carbon matrix without introducing any other heteroatoms. To the best of our knowledge, the outstanding performance of VFPC is not only better than those of other porous pure carbon materials as provided in the literatures (Table S1), but also comparable with other kinds of carbon-based anode materials like carbon nanotubes, heteroatom-doped porous carbon, graphene and their composites, etc^35^,^36^,^37^.

The FPC and VFPC in fully lithium intercalation state (denoted as Li-FPC and Li-VFPC, respectively) were also investigated by SAXS method. The Porod analysis of both Li-FPC and Li-VFPC shows a negative deviation (Fig. S6), indicating the existence of a transition zone, i.e., an interface layer, between two phases without sharp boundary^32^,^38^. The values of average thickness of the transition zone are 1.97 and 2.37 nm for Li-FPC and Li-VFPC, respectively, which were determined by the correction process of negative deviation. Considering the absence of micropores in both Li-FPC and Li-VFPC (insets in Fig. 4f), we speculate that all the micropores are fully filled by lithium atoms, as the electrolyte molecules are too large to embed into such small micropores. Therefore, we consider that the transition zone is comprised of solid electrolyte interface (SEI) film and surface defects filled by lithium, while the bulk phase is comprised of lithium carbides and lithium-embeded micropores. The electron densities (ρ_e_) of the two phases and the transition zone can be calculated from Porod constant (see Supplementary SI6)^38^. As the scattering background has been removed, the electron density of the pore phase can be treated as zero, and the electron density difference of the two phase can be regarded as the electron density of the bulk phase (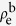). By comparing with other reported electrochemical lithium intercalation carbon products (see Supplementary SI6 and Table S2), the 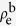 of Li-FPC is 1.101 mol cm^−3^ which can be deemed as a bulk storage with x = 1–1.5 in Li_x_C_6_, and 1.132 mol cm^−3^ of Li-VFPC can be deemed as a bulk storage with x = 1.5–2 in Li_x_C_6_. The average ρ_e_ of transition zones are 0.551 and 0.566 mol cm^−3^ for Li-FPC and Li-VFPC, respectively, and these low ρ_e_ might be ascribed to the surface storage and defect storage of lithium atoms. It can be seen that the higher surface fractal dimension and the larger closed pore volume can lead to more lithium storage in both surface and bulk storage modes.

From the data analysis above, it can be found that both of the fractal structures and the pores that cannot be detected by N_2_ sorption method have played an dramatic role in lithium storage in carbon materials. According to the theory of electrochemical reaction on fractal surface, surface or interface with higher surface fractal dimension can provide larger electrochemical active surface area and smaller internal resistance^39^,^40^. The VFPC with a higher surface fractal dimension exhibits a better electrochemical performance due to the enhanced surface storage mode that has been proved by the transition zone analysis using SAXS method. The electrochemical impedance spectra (EIS) analysis of FPC and VFPC have also shown that higher surface fractal dimension leads to a lower charge transfer resistance (see Fig. S7 and Table S3). The electrochemical reactions occurring on the surface and interface were also strengthened by the larger ΔS and D_s_, which led to a stronger peak in CV plot at around 0.7 V as can be seen in Fig. 5a,b. As the surface and interface with higher D_s_ can provide higher electrochemical active site density, the larger specific surface area will provide much more active sites for the lithiation reactions. This view point is also proved by the EIS results with a lower charge transfer resistance indicating a better ionic diffusion in VFPC (see Table S3). The surface storage of Li in carbon was proved by TEM results which show a uniform dispersion of Li_2_O, and the statistical results also illustrate the advantage of surface fractal structure (see Supplementary SI8 and Fig. S8). The morphology images of FPC and VFPC after 50 charge-discharge cycles show the stabilizing effect of surface fractal structure by displaying a more smooth surface of VFPC (see Fig. S9). If we regard ΔS and ΔV_mic_ as specific surface area and micropore volume that cannot be detected by N_2_ sorption method, it can be found that VFPC prepared by vacuum pyrolysis can provide a larger ΔS and ΔV_mic_, which finally achieve an ultrahigh lithium storage performance. After lithium intercalation, the micropores disappeared (Fig. 4f), and the S_SAXS_ of FPC and VFPC fell to 110 and 103 m^2^ g^−1^, respectively, which could be attributed to the filling of micropores by lithium atoms and the formation of the relatively thick transition zones. In addition, the higher intercalation degree of bulk storage in VFPC is also illustrative of the important roles of surface fractal structures, ΔS and ΔV_mic_ in promoting the lithium storage performance by different storage modes.

## Conclusion

In conclusion, the fractal structures of porous carbon, as well as the pores that cannot be detected by N_2_ sorption method, advance the lithium storage capacity dramatically by enhancing the bulk storage, surface storage and defect storage. The vacuum pyrolysis process can induce the formation of multi-fractal structure of porous carbon and more closed pores. The macropores and mesopores offer short path of lithium ions by optimizing the conditions for electrolyte penetration. Subnanopores distributed with fractal structures can provide a large amount of lithium storage active sites, which may generate a transition zone rich in lithium atoms. We believe that the idea of the fractal structures and the closed pore storage mode can be introduced into the design of electrodes for other metal-ion batteries and energy storage devices.

## Methods

### Synthesis of Zn-MOF and its derived porous carbon

The Zn-MOF was synthesized according to the procedure described in previous report^20^. In a typical synthesis process, 2.3 g of benzene-1,4-dicarboxylic acid and 11.9 g of zinc nitrate hexahydrate were dissolved into 160 ml dimethyl formamide (DMF), then transferred into a Teflon-lined stainless steel autoclave. It was then sealed and maintained at 120 °C for 24 h. After filtration and washing by DMF, the Zn-MOF was collected and dried under vacuum at 150 °C overnight. The precursor Zn-MOF was pyrolyzed at 1000 °C in N_2_ under atmospheric pressure and vacuum (the furnace pressure was kept in 0.02–0.04 MPa) to obtain the products, respectively. The sample pyrolyzed under the nitrogen atmosphere at room pressure is named as FPC, and the sample prepared by the vacuum carbonization is marked as VFPC.

### Materials characterization

Field-emission scanning electron microscopy (FE-SEM; ZEISS SUPRATM 55 field emission microscope), energy-dispersive X-ray spectroscopy(EDS; attached to SEM, Oxford Instruments), X-ray diffraction (XRD; Rigaku D/max-2500B2+/PCX system with CuKα = 1.5406 Å, 2θ = 5–90°) and Raman spectroscopy using a 532 nm laser (Aramis, Jobin Yvon) were carried out to investigate the structure and composition of the samples. Nitrogen sorption isotherms were measured with ASAP2020 (Micromeritics, USA) with a degassed process at 300 °C for 6 h. Specific surface areas were obtained according to the Brunauer-Emmet-Teller (BET) model, and pore size distributions were simulated by the Non-local density functional theory (NLDFT) model. The total pore volume was calculated from the amount adsorbed at P/P_0_ = 0.994.

SAXS experiment was performed using synchrotron radiation as X-ray source at 1W2A station at Beijing Synchrotron Radiation Facility (BSRF). The focused X-ray beam size was about 1.4 × 0.2 mm^2^ with a flux of 5.5 × 10^11^ phs s^−1^ at 2.5 GeV and 250 mA. Incident X-ray wavelength λ was 0.154 nm. SAXS images were recorded with a Mar 165 CCD detector. The detected intensity is a function of the modulus of the scattering vector q = 4πsinθ/λ, where 2θ is the scattering angle, and the obtained results were smeared SAXS data. The calibration of scattering vector was performed involving the incident X-ray wavelength and the sample to detector distance (1608 mm). The normalization and the removal of background were possessed by I = I_s_ − (K_s_/K_b_)I_b_, where I is the processed scattering intensity after background subtraction; I_s_ and I_b_ are scattering intensity of sample and background, respectively; K_s_ and K_b_ are the transmitted X-ray intensity of the sample and the background, respectively.

### Electrochemical measurements

Electrochemical performances were measured by using 2032 coin-type cells with lithium foil as counter electrode. The working electrodes were prepared by pasting the mixtures of 80% active mass, 10% acetylene black and 10% poly (vinylidene difluoride) onto foam nickel. The loading mass of active materials in one working electrode is 2 mg. The electrolyte was a 1 M LiPF_6_ solution in a mixture of ethylene carbonate/dimethyl carbonate (1:1 v/v). The galvanostatical charge-discharge tests were carried out within the potential range of 0.01–3 V vs. Li/Li^+^ at various rates. Electrochemical impedance spectroscopy(EIS) and cyclic voltammetry (CV) measurements of the samples were preformed on an electrochemical workstation (CHI 660B). EIS tests were obtained by applying a sine wave with an amplitude of 5.0 mV over the frequency range from 100 kHz to 0.01 Hz. The cyclic voltammograms were obtained over the potential range of 0.01–3 V vs. Li/Li^+^ at a scan rate of 0.1 mV s^−1^.

## Additional Information

**How to cite this article**: Li, A. *et al*. MOF-derived multifractal porous carbon with ultrahigh lithium-ion storage performance. *Sci. Rep.* **7**, 40574; doi: 10.1038/srep40574 (2017).

**Publisher's note:** Springer Nature remains neutral with regard to jurisdictional claims in published maps and institutional affiliations.

## Supplementary Material

Supplementary Information

## Figures and Tables

**Table 1:** The porous properties from SAXS and N2 sorption experiments for FPC and VFPC.

Materials	S_BET_^a^	V^b^	V_mic_^c^	S_SAXS_	V_SAXS_	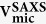	ΔS^d^	ΔV_mic_^d^
FPC	2565	2.76	0.14	2826	3.91	0.41	261	0.27
VFPC	2587	2.85	0.09	3125	3.82	0.40	538	0.31

N_2_ sorption method: S_BET_-specific surface area (m^2^ g^−1^); V - total pore volume (cm^3^ g^−1^); V_mic_-micropore volume (cm^3^ g^−1^).

SAXS method: S_SAXS_-specific surface area (m^2^ g^−1^) ; V_SAXS_ - total pore volume (cm^3^ g^−1^); VSAXS mic-micropore volume (cm^3^ g^−1^).

^a^Calculated by BET method.

^b^Calculated from the adsorbing capacity when P/P_0_ = 0.995.

^c^Calculated by t-plot method.

^d^ΔS = S_SAXS_ − S_BET_, (m^2^ g^−1^); ΔV_mic_ = 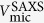 − V_mic_, (cm^3^ g^−1^).
