# Supplementary material for: MOF-derived multifractal porous carbon with ultrahigh lithium-ion storage performance
Source: Sci Rep. 2017 Jan 11;7:40574. doi: 10.1038/srep40574 (PMC5225442; doi:10.1038/srep40574)
Supplement: Supplementary Information [file srep40574-s1.pdf]

Supplementary Information

MOF-derived multifractal porous carbon with ultrahigh lithium-ion storage performance

Ang Li[1], Yan Tong[1], Bin Cao[1], Huaihe Song[1]*, Zhihong Li[2], Xiaohong Chen[1], Jisheng Zhou[1], Gen Chen[3], Hongmei Luo[3]

1. State Key Laboratory of Chemical Resource Engineering, Beijing Key Laboratory of Electrochemical Process and Technology for Materials, Beijing University of Chemical Technology, Beijing, 100029, P. R. China.
2. Beijing Synchrotron Radiation Facility, Institute of High Energy Physics, Chinese Academy of Sciences, 19B Yuquan Road, Beijing 100049, P. R. China.
3. Department of Chemical and Materials Engineering, New Mexico State University, Las Cruces, New Mexico 88003, United States.
*Corresponding Author: E-mail: songhh@mail.buct.edu.cn.

SI.1 Characterization of Zn-MOF

In Figure S1a, the XRD pattern of Zn-MOF exhibits a high crystallinity, which is similar to the reference report[s1]. The nitrogen adsorption isotherms of Zn-MOF display characteristics of type Ⅰ (Fig. S1b) with a rapid uptake of adsorption at low relative pressure ($P/P_0 < 0.1$), indicating that the pores in Zn-MOF are micropores[s2]. The type of the pores is further confirmed by the pore size distribution calculated using the non-local density functional theory (NLDFT) model (inset of Fig. S1b). The specific surface area from BET method is also obtained to be 2137 $m^2\ g^{-1}$. The total pore volume of Zn-MOF calculated from the amount adsorbed at $P/P_0 = 0.994$ is 1.15 $cm^3\ g^{-1}$.

The optical microscope images were taken by the polarized-light microscope (Olympus BX51M), as illustrated in Fig. S1c&d.

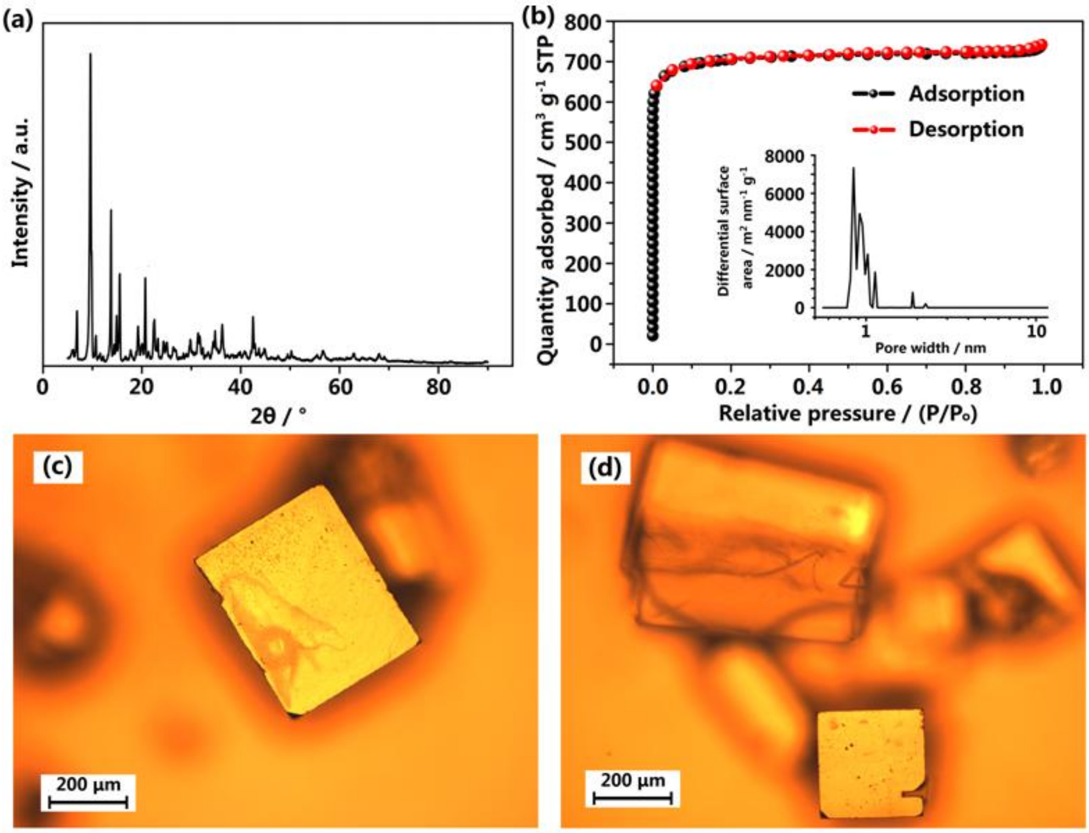

Figure S1. Structure and morphology characterization of Zn-MOF. (a) XRD pattern of Zn-MOF. (b) nitrogen adsorption–desorption isotherms of Zn-MOF, and the inset is pore size distribution. (c) and (d) are the optical microscope images of the Zn-MOF crystals.

SI.2 Structure and composition of FPC and VFPC

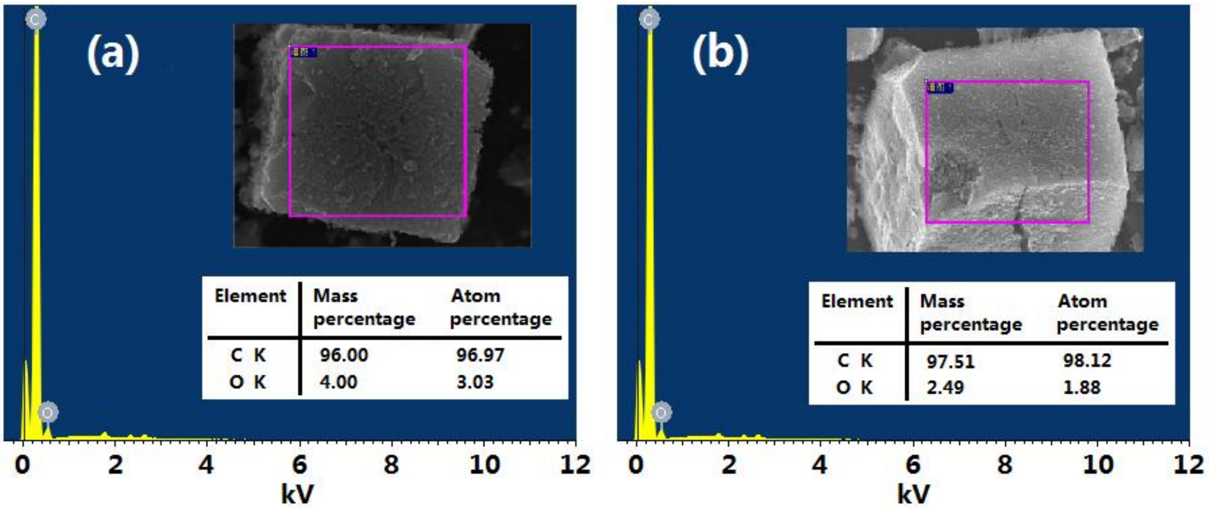

Figure S2. Composition characterization of FPC and VFPC. (a) and (b) are EDS spectra of FPC and VFPC respectively. VFPC shows higher carbon purity than FPC, indicating that the vacuum pyrolysis can help to improve the purity of carbon element.

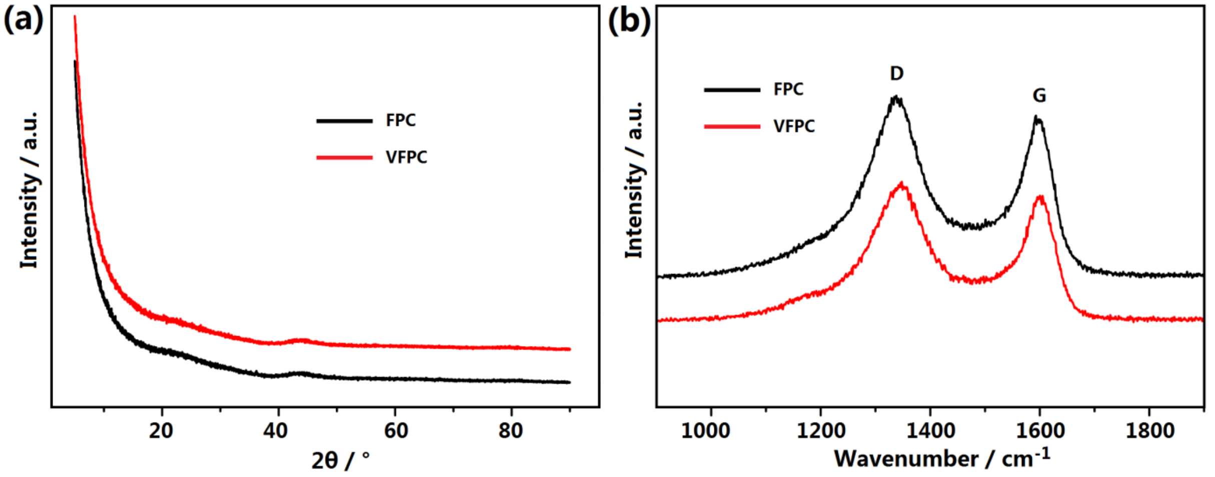

Figure S3. Crystalline and structural characterization of FPC and VFPC: (a) XRD patterns and (b) Raman spectra.

SI.3 Porous property characterization of FPC and VFPC by SAXS

Porod's law is one of the basic theories in SAXS, which can be used to describe the porous properties of the samples[s3]. In Fig. S4a&b, the Porod plots of both FPC and VFPC show a positive deviation, indicating a quasi two-phase system with micro-fluctuations of electron density within any phase of a two-phase system. For the positive deviation from the Porod's law, one obtains[s3]:

$$I(q) = \frac{K}{q^3}\exp(bq^2) \quad \text{or} \quad \ln[q^3 I(q)] = \ln K + bq^2 \qquad (1)$$

where I(q) is the scattering intensity, q is the scattering vector, $q = 4\pi\sin\theta/\lambda$, 2θ is the scattering angle, K is the Porod constant, and b is a constant related to the size of the regions with

micro-fluctuations of electron density. The fitting results show that K for FPC and VFPC are 103.5 and 280.0, respectively.

The specific surface area of the scatterers can be calculated by Porod method[s3]:

$$S_V = \pi P(1-P)\frac{K}{Q} \qquad (2)$$

where S_V is the total surface per unit of volume, P is the porosity of the sample, and Q is the invariant constant, which is given by[s3]:

$$Q = \int_0^\infty qI(q)dq \qquad (3)$$

The SAXS method can also be used to simulate the scatterer size distribution. The scattered intensity I(q) for a polydisperse system, which can be expressed by[s3,s4]:

$$I(q) = C\int_0^\infty D_V(r)r^3 I_O(q,r)dr \qquad (4)$$

where C is a constant, $D_V(r)$ is defined as the volume distribution of the scatterer with size r. $I_o(q,r)$ is the scattering intensity of the radially symmetric scatterer of size r. The determination of $D_V(r)$ is the key to calculate the pore size distribution, and we used the cascade tangent rule of Jellinek method based on Guinier's approach (Fig. S4c&d). The mean size of scatterers was calculated by:

$$\bar{r} = \sum r_i D_{v_i} \qquad (5)$$

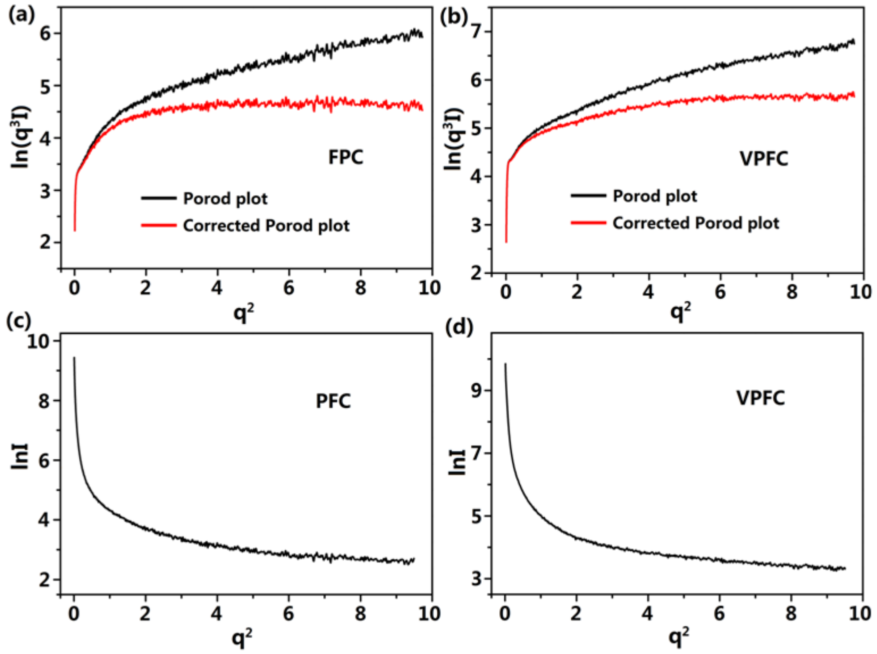

Figure S4. SAXS data analysis of FPC and VFPC. Porod analysis and positive deviation corrections of (a) FPC and (b) VFPC, respectively. Guinier plots of (c) FPC and (d) VFPC, respectively.

SI.4 Calculation of micropore volume of FPC and VFPC by SAXS[s5]

Usually, the micropore volume V_{mic} can be calculated from the density of carbon phase with micropores dispersed in it, eg. the microporous backbone phase ρ_{mc} and the density of amorphous carbon phase ρ_c. The relationship of the parameters can be expressed by:

$$V_{mic} = \rho_{mc}^{-1} - \rho_c^{-1} \qquad (6)$$

In order to obtain the ρ_{mc}, the bulk can be treated as a two-phase system consisting of microporous backbone phase and the macro/meso-pore scatterers phase; thus, the relationship between the macroscopic density ρ and the microporous backbone phase ρ_{mc} can be expressed as:

$$\rho = \rho_{mc} - \frac{Q}{2\pi^2 C^2} \qquad (7)$$

where the constant C = 8.504 ×10^{11} m kg^{-1} that connects the mass density of the scattering entities to the scattering cross section.

SI.5 Fractal characterization of FPC and VFPC by SAXS[s3]

SAXS method has been widely used to investigate the fractal characteristics of the structure of irregular objects. Briefly in the theory of small-angle X-ray scattering, the SAXS intensity from fractal objects has a power-law form:

$$I(q) = I_0 q^{-\alpha} \qquad (8)$$

where I_0 and α are constants. It should be point out that equation 8 are established only when the value of q satisfies the inequality $q\xi \gg 1$, where ξ corresponds to the scale of the structure of the scatterers.

For porous fractals, the porous fractal dimension D_p is given by:

$$D_p = \alpha, \quad 1 < \alpha < 3 \qquad (9)$$

whereas for surface fractals, the surface fractal dimension D_s is given by:

$$D_s = 6 - \alpha, \quad 3 < \alpha < 4 \qquad (10)$$

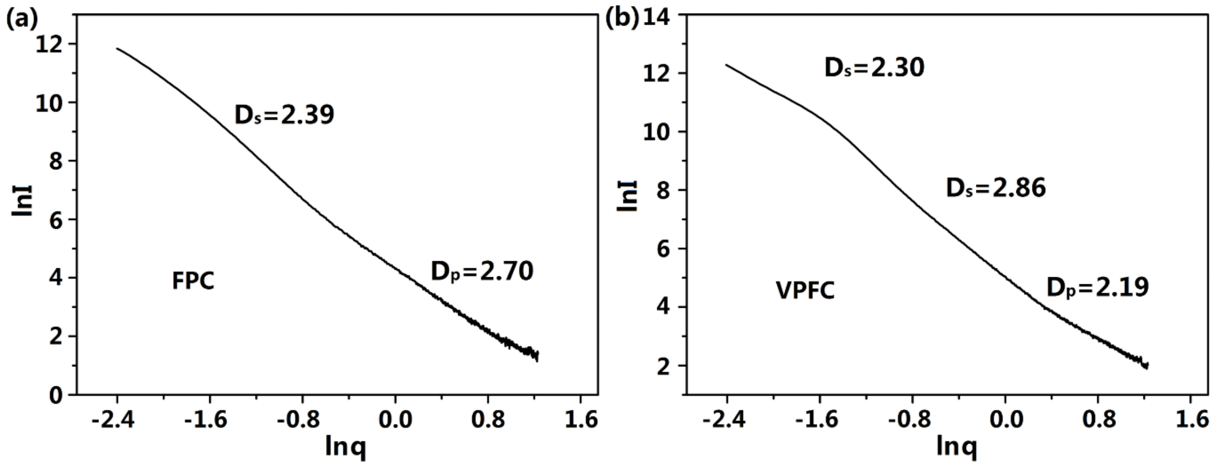

Figure S5. Fractal structures characterization of FPC and VFPC. (a)lnI-lnq plots of FPC, which show two linear domains, corresponding to the surface fractal and porous fractal behaviors. (b) lnI-lnq plots of VFPC, and the curves display three linear domains which can be ascribed to two surface fractal areas and a porous fractal.

SI.6 Structure characterization of Li-FPC and Li-VFPC by SAXS

For the negative deviation from the Porod's law, the correction form can be written as[s3]:

$$I(q) = \frac{K}{q^3}\exp(-\sigma^2 q^2) \quad \text{or} \quad \ln[q^3 I(q)] = \ln K - \sigma^2 q^2 \qquad (11)$$

where σ is the standard deviation of the Gaussian smoothing function, which is a parameter related to the thickness of the transition zone. The thickness of the transition zone E can be expressed by[s3]:

$$E = (2\pi)^{0.5}\sigma \qquad (12)$$

The electrical density of the scatterers can also be obtained from SAXS, and the electrical density is related to K by the equation[s6]:

$$K = \pi^2 S \Delta\rho_e^2 \qquad (13)$$

where S is the surface per unit weight, $\Delta\rho_e$ is the contrast in electron density between the two phases.

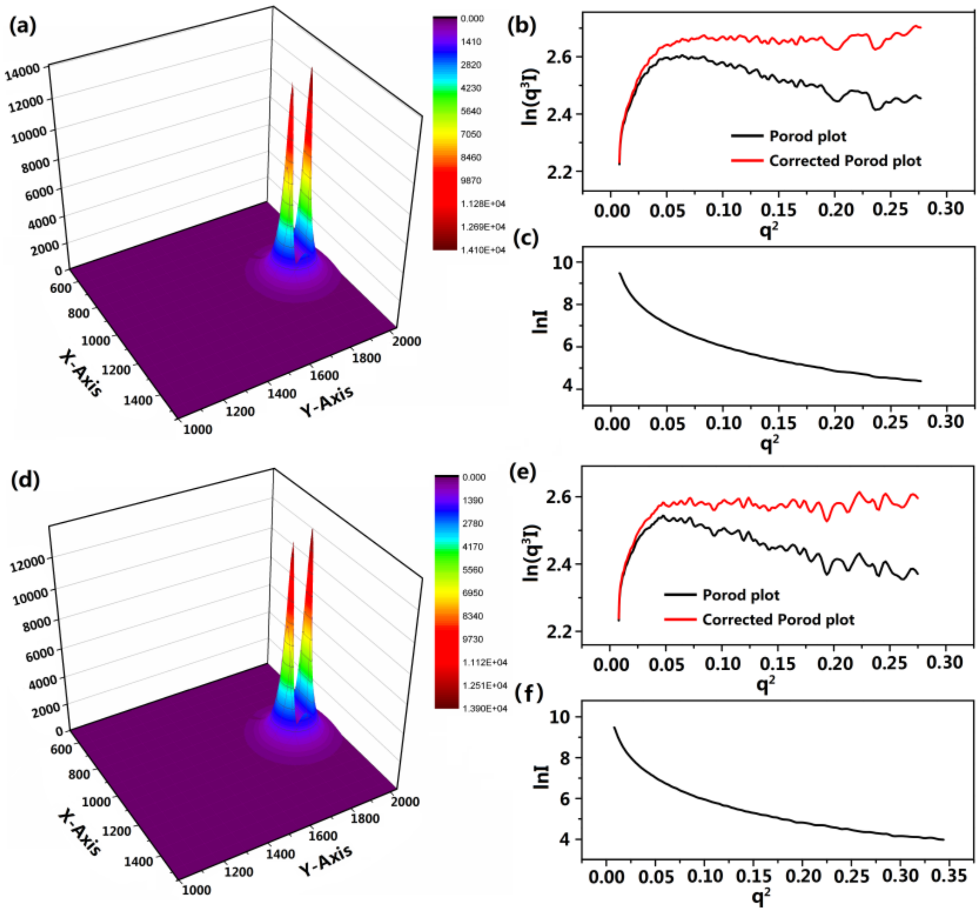

Figure S6. Structure characterization and pore size distribution determination of Li-FPC and Li-VFPC. The data and analysis of Li-FPC: (a) 2D SAXS pattern with transmitted intensity as a Z-axis; (b) Porod plots and its negative deviation correction; (c) Guinier plots. The data and analysis of Li-VFPC: (d) 2D SAXS pattern with transmitted intensity as a Z-axis; (e) Porod plots and its negative deviation correction; (f) Guinier plots. The color bars in (a) and (d) are the scaling of intensity.

SI.7 Electrochemical impedance spectra (EIS) results of FPC and VFPC

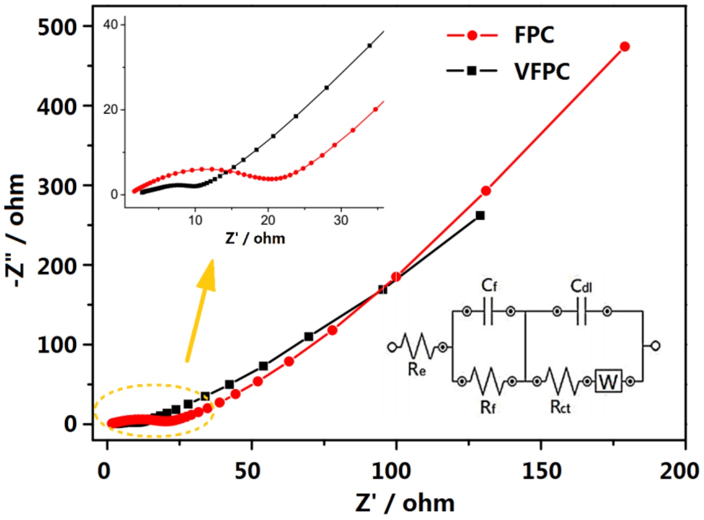

Figure S7. EIS results of FPC and VFPC.

SI.8 TEM images of FPC and VFPC in full lithium insertion state

As illustrated in HRTEM mode, large amounts of nanoparticles dispersing in carbon matrix densely can be seen in Fig. S8a&d. Detail analysis of the HRTEM images show that these nanoparticles are composed of Li_2O in Fig. S8b&e, the areas in yellow circles corresponded to Li_2O (220) fringes with a interplanar spacing of 0.163 nm in Fig. S8b, and Li_2O (111) fringes with 0.258 nm in Fig. S8e. Li_2O is the products formed during the lithiation process, of which the distributing appearance can reflect the reactivity of the electrode materials. In order to observe the difference of the distributing appearance of Li_2O in FPC and VFPC intuitively, the EFTEM mode was used. In EFTEM mode, the Li_2O particles appear as white spots in the images (Fig. S8c&f). The Li_2O phase dispersing in VFPC is more uniform and smaller than those in FPC, and the average size of nanoparticles is 2.13 nm and 1.32 nm for FPC and VFPC, respectively. Besides, the size statistical results of Li_2O nanoparticles in carbon matrix are displayed as insets in Fig. S8a&d. The Li_2O dispersing in the VFPC carbon matrix shows a more narrow distribution in size than that of FPC.

Besides the intercalation of Li into graphene layers, the formation of Li_2O during discharge process (Li-insertion) can be regarded as reaction product of lithium storage on the interface of electrode/electrolyte. Therefore, the nucleation process of Li_2O can be used to reflect the dispersion of Li on electrode materials. The results in Fig. S8 indicate that the lithiation reactions occurred in VFPC exhibit an excellent homogeneity and a high reactivity.

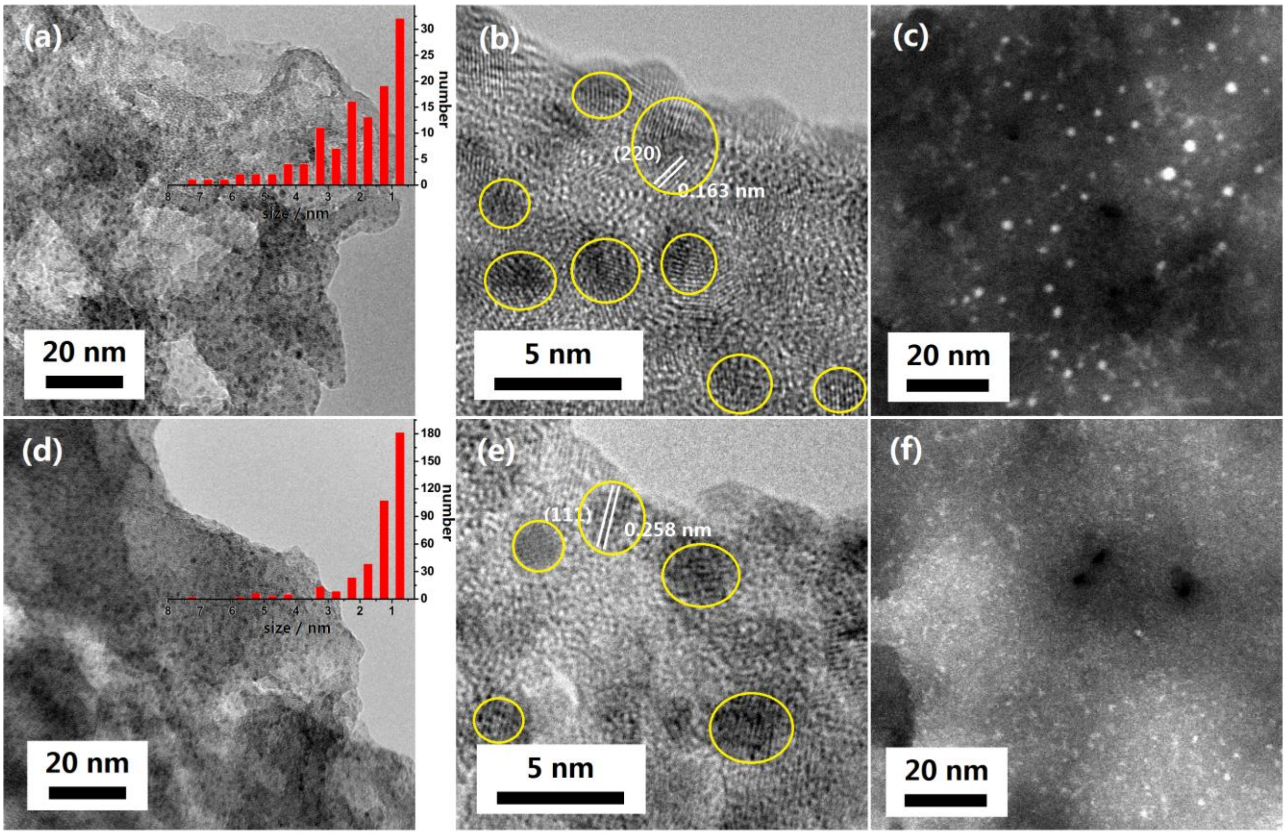

Fig.S8 The TEM images for FPC and VFPC. (a&b) HRTEM and (c) EFTEM for FPC; (d&e) HRTEM and (f) EFTEM for VFPC. The insets in (a&d) are the statistical results of Li_2O particles in FPC and VFPC, respectively.

SI.9 SEM images of FPC and VFPC after 50 charge-discharge cycles

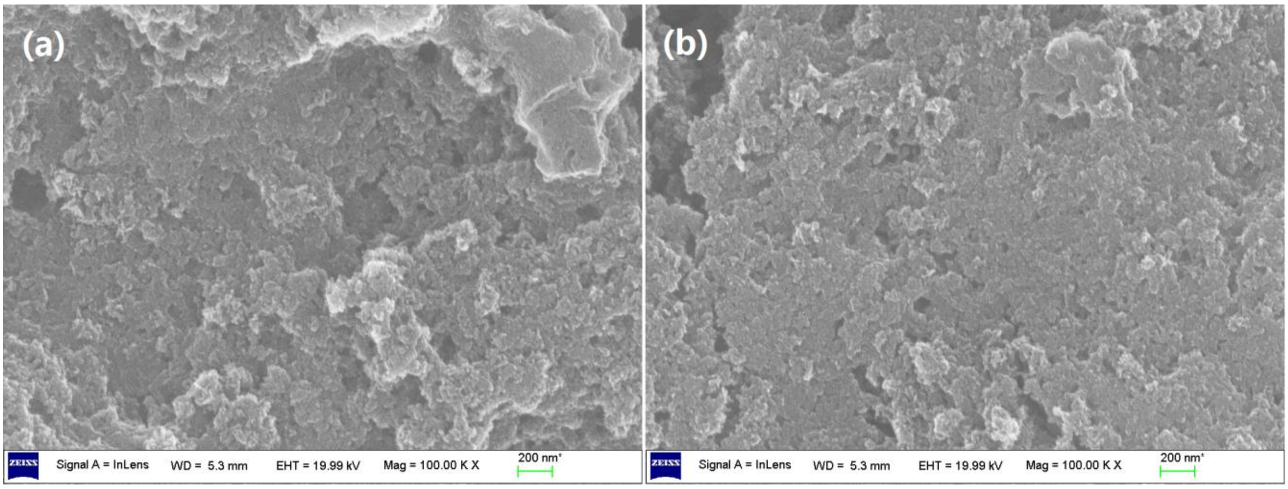

Fig.S9 The SEM images for FPC and VFPC after 50 cycles.

Materials	Current density (mAg^{-1})	Discharge specific capacity ($mAhg^{-1}$)	Cycle number	Ref.
PGr	100	833	25	s7
OMC	100	850	20	s8
OMC	100	876	100	s9
OMC	50	500	50	s10
MC-CNTs	0.1C	786	20	s11
PCNFs	50	454	10	s12
PCNFs	100	1132	100	s13
PCNFs	50	435	50	s14
HPC	20	748	50	s15
HPC	167.4	192	20	s16
HPC	1C	270	100	s17
HPC	0.2C	500	40	s18
HPC	50	995	50	s19
HPC	100	799	80	s20
HPC	500	506	50	s21
	1000	355	50	
HPC	50	480	70	s22
**FPC**	**0.2C**	**1470**	**50**	**This work**
	**2C**	**820**	**20**	
**VFPC**	**0.2C**	**2016**	**50**	**This work**
	**2C**	**1012**	**200**	

Table S1. The specific capacity values of porous pure carbon materials in previous literature and this paper.

PGr - porous grahene; OMC - ordered mesoporous carbon; MC-CNTs - mesoporous carbon-carbon nanotubes; PCNFs - porous carbon nanofibers; HPC - Hierarchical porous carbon

		mass density/g cm^{-3}	electron density/mol cm^{-3}	Ref.
Li		0.534	0.231	na
LiC_6		2.211	1.092	s23
LiC_4		2.271	1.115	s23
Li_2C_6		2.362	1.154	s23
Li_3C_6		2.517	1.219	s23
lithiated phase	1000	na	1.101	this work
	1000vac	na	1.132	
$LiPF_6$		1.5	0.691	s24
LiF		2.635	1.219	s24
Li_2CO_3		2.11	1.028	s24
CH_3OCO_2Li		1.26	0.802	s24
PEO		1.15-1.26	0.627-0.687	s24
$OP(OCH_3)_3$		1.215	0.625	s24
transition zones	1000	na	0.551	this work
	1000vac	na	0.566	

Table S2. The electron densities of some lithium carbides and components of SEI films.

Sample	R_f (Ω)	R_{ct} (Ω)
FPC	4.35	15.49
VFPC	5.04	10.44

Table S3. Kinetic parameters of NWAs and RT-NWAs electrodes after 30 cycles.

Reference

s1. Banerjee, A. *et al.* MOF-Derived Crumpled-Sheet-Assembled Perforated Carbon Cuboids as Highly Effective Cathode Active Materials for Ultrahigh Energy Density Li-Ion Hybrid Electrochemical Capacitors (Li-HECs). *Nanoscale,* **6**, 4387-4394 (2014).

s2. Chaikittisilp, W. *et al.* Nanoporous Carbons through Direct Carbonization of a Zeolitic Imidazolate Framework for Supercapacitor Electrodes. *Chem. Commun*. **48**, 7259-7261 (2012).

s3. Li, Z. H. A Program for SAXS Data Processing and Analysis. *Chin. Phys. C*, **37**, 108002 (2013).

s4. Jellinek, M. H., Soloman, E., Fankuchen, I. I. Measurement and Analysis of Small-Angle X-Ray Scattering. *Ind. Eng. Chem. Anal. Ed*. **18**, 172-175 (1946).

s5. Reichenauer, G. Micropore Adsorption Dynamics in Synthetic Hard Carbons. *Adsorption*, **11**, 467-471 (2005).

s6. Deganello, G. *et al.* Structure of Natural Water-Containing Glasses from Lipari (Italy) and Eastern Rhodopes (Bulgaria): SAXS, WAXS and IR studies. *J. Non-Cryst. Solids*. **232-234**, 547-553 (1998).

s7. Fang, Y. *et al.* Two-Dimensional Mesoporous Carbon Nanosheets and their Derived Graphene Nanosheets: Synthesis and Efficient Lithium Ion Storage. *J. Am. Chem. Soc.* **135**, 1524-1530 (2013).

s8. Zhou, H., Zhu, S., Hibino, M., Honma, I., Ichihara, M. Lithium Storage in Ordered Mesoporous Carbon (CMK-3) with High Reversible Specific Energy Capacity and Good Cycling Performance. *Adv. Mater*. **15**, 2107-2111 (2003).

s9. Kim, M. S. *et al.* Morphology-Dependent Li Storage Performance of Ordered Mesoporous Carbon as Anode Material. *Langmuir,* **29**, 6754-6761 (2013).

s10. Li, H. Q., Liu, R. L., Zhao, D. Y., Xia, Y. Y. Electrochemical Properties of an Ordered Mesoporous Carbon Prepared by Direct Tri-Constituent Co-Assembly. *Carbon*, **45**, 2628-2635 (2007).

s11. Guo, B. *et al.* Soft-Templated Mesoporous Carbon-Carbon Nanotube Composites for High Performance Lithium-Ion Batteries. *Adv. Mater*. **23**, 4661-4666 (2011).

s12. Ji, L., Lin, Z., Medford, A. J., Zhang, X. Porous Carbon Nanofibers from Electrospun Polyacrylonitrile/SiO_2 Composites as an Energy Storage Material. *Carbon*, **47**, 3346-3354 (2009).

s13. Xing, Y., Wang, Y., Zhou, C., Zhang, S., Fang, B. Simple Synthesis of Mesoporous Carbon Nanofibers with Hierarchical Nanostructure for Ultrahigh Lithium Storage. *ACS Appl. Mater. Inter*. **6**, 2561-2567 (2014).

s14. Ji, L., Zhang, X. Fabrication of Porous Carbon Nanofibers and Their Application as Anode Materials for Rechargeable Lithium-ion Batteries. *Nanotechnology*, **20**, 155705 (2009).

s15. Song, R. *et al.* Hierarchical Porous Carbon Nanosheets and Their Favorable High-Rate Performance in Lithium Ion Batteries. *J. Mater. Chem.* **22**, 12369-12374 (2012).

s16. Yi, J. *et al.* Preparation of Hierarchical Porous Carbon and Its Rate Performance as Anode of Lithium Ion Battery. *J. Power Sources*, **196**, 6670-6675 (2011).

s17. Etacheri, V., Wang, C., O'Connell, M. J., Chan, C. K., Pol, V. G. Porous Carbon Sphere Anodes for Enhanced Lithium-ion Storage. *J. Mater. Chem. A,* **3**, 9861-9868 (2015).

s18. Hu, Y. S. *et al.* Synthesis of Hierarchically Porous Carbon Monoliths with Highly Ordered Microstructure and Their Application in Rechargeable Lithium Batteries with High-Rate capability. *Adv. Funct. Mater*. **17**, 1873-1878 (2007).

s19. Xiao, J. *et al.* Facile Synthesis of Hydrogenated Carbon Nanospheres with a Graphite-like Ordered Carbon Structure. *Nanoscale*, **5**, 11306-11312 (2013).
s20. Fang, B., Kim, M. S., Kim, J. H., Lim, S., Yu, J. S. Ordered Multimodal Porous Carbon with Hierarchical Nanostructure for High Li Storage Capacity and Good Cycling Performance. *J. Mater*. *Chem*. **20**, 10253-10259 (2010).
s21. Song, R. *et al.* Effects of Copper Nitrate Addition on The Pore Property and Lithium Storage Performance of Hierarchical Porous Carbon Nanosheets from Phenolic Resin. *Electrochim. Acta*, **127**, 186-192 (2014).
s22. Wang, F. *et al.* Simple Synthesis of Novel Hierarchical Porous Carbon Microspheres and their Application to Rechargeable Lithium-Ion Batteries. *Carbon,* **81**, 314-321 (2015).
s23. Doh, C-H., Han, B. C., Jin, B-S., Gu, H-B. Structures and Formation Energies of Li_xC_6 (x = 1-3) and its Homologues for Lithium Rechargeable Batteries. Bull. *Korean Chem. Soc*. **32**, 2045-2050 (2011).
s24. Leroy, S. *et al.* Surface Film Formation on A Graphite Electrode in Li-Ion Batteries: AFM and XPS Study. *Surf. Interface Anal*. **37**, 773-781 (2005)
